# QSPcc reduces bottlenecks in computational model simulations

**DOI:** 10.1038/s42003-021-02553-9

**Published:** 2021-09-01

**Authors:** Danilo Tomasoni, Alessio Paris, Stefano Giampiccolo, Federico Reali, Giulia Simoni, Luca Marchetti, Chanchala Kaddi, Susana Neves-Zaph, Corrado Priami, Karim Azer, Rosario Lombardo

**Affiliations:** 1 Fondazione the Microsoft Research, University of Trento Centre for Computational and Systems Biology, Rovereto, Italy; 2 Data and Data Science – Translational Disease Modeling, Sanofi, Bridgewater, NJ USA; 3 Present Address: Department of Computer Science, University of Pisa, Pisa, Italy; 4 Present Address: Axcella Health, Cambridge, MA USA

**Keywords:** Computational models, Computer modelling, Drug discovery

## Abstract

Mathematical models have grown in size and complexity becoming often computationally intractable. In sensitivity analysis and optimization phases, critical for tuning, validation and qualification, these models may be run thousands of times. Scientific programming languages popular for prototyping, such as MATLAB and R, can be a bottleneck in terms of performance. Here we show a compiler-based approach, designed to be universal at handling engineering and life sciences modeling styles, that automatically translates models into fast C code. At first QSPcc is demonstrated to be crucial in enabling the research on otherwise intractable Quantitative Systems Pharmacology models, such as in rare Lysosomal Storage Disorders. To demonstrate the full value in seamlessly accelerating, or enabling, the R&D efforts in natural sciences, we then benchmark QSPcc against 8 solutions on 24 real-world projects from different scientific fields. With speed-ups of 22000x peak, and 1605x arithmetic mean, our results show consistent superior performances.

## Abstract (web summary)

Lombardo and colleagues present QSPcc, a computational code compiler designed to convert code from popular scientific programming languages, such as MATLAB or R, into fast-running C code. This reduces the computational load required for complex modelling approaches and reduces user investment learning additional complex languages.

## Introduction

In Natural Sciences, modeling often involves the development of a mathematical formulation of the time evolution of physical entities, typically using ordinary differential equations (ODEs). ODEs are a deterministic representation of the underlying structure of the considered system. ODE models are used in physics, chemistry, biology, engineering, economics and many other application fields. Among these fields, quantitative systems pharmacology (QSP) is an emerging discipline, consisting in an approach to describe pathophysiology of a disease and the response to pharmacological intervention^1,2^. In the context of systems biology, ODEs depict the dynamical properties of the interaction between the drug and the biological system as a whole^3^, an approach that can help the design and the validation of non-clinical and clinical experiments and can accelerate drug discovery and development^4,5^.

Finding solutions to the initial value problem (IVP) is a task that, apart for a limited number of cases, must be performed numerically^4,6,7^. To this aim, different ODE integration algorithms have been developed. Their implementations are available in mathematical libraries of most programming languages. However, not all implementations have the same efficiency in terms of accuracy and integration time. Many different integration methods can be employed, each of them with its own parameter set. Models may not necessarily involve ODEs and may use other algorithmic scripting code. Time performance of different programming languages is also an issue. With increasing system size in terms of multi-scale systems, number of simulated variables, length of simulated time, and numerical stiffness, execution time can increase up to the point of making the problem intractable. QSP models can contain multiple biological scales and large sets of simulated molecules, that may require years of simulated lifetime and repeated runs for virtual patient populations. High-level and easy-to-use interpreted languages such as MATLAB^8^ and R^9^ tend to be slower in terms of execution time than lower-level and compiled languages such as C and Fortran. However, the former usually offer a more user-friendly environment for the development of models and are more familiar among scientists working in different research fields, while the latter requires programming skills that are usually beyond the average scientist’s knowledge and are prone to diverting the research efforts from the modeling activity to coding and debugging.

The elastic mesh bridging the five pillars of biological reasoning, modeling, performance issues, ease-of-use and flexibility has been woven by multiple solutions, each focusing on specific areas. Facing the computational complexity of large mathematical models in rare lysosomal storage disorders (LSDs), in this paper, we review and systematically benchmark the currently available solutions allowing to cut down the increasing simulation time of the modeled pathophysiology. We also contribute a compiler-based solution, namely QSPcc, delivering *flexibility* in the variety of handled models, *ease-of-use* in the limited or not-required adaptations to existing code and *performance speedup* in large scale projects.

## Results

**Fig. 1:**
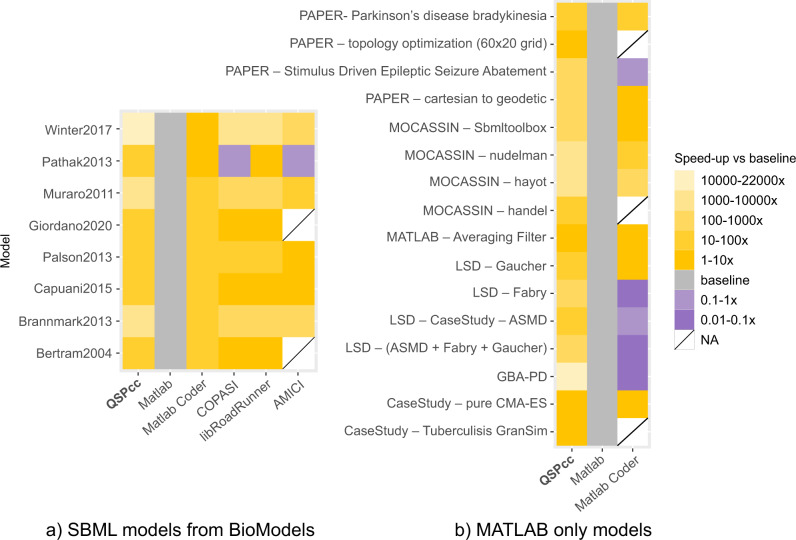
QSPcc consistently delivers superior performances of different orders of magnitude compared to other solutions. The gradient violet to yellow depicts how many times the given combination model/translator is faster than MATLAB (the lighter, the better). The running time of each model was measured averaging five different executions. White blocks represent models that cannot be translated to C even after a significant hands-on effort (see Comparison with other tools for further details). **a** Comparison of 5 SBML-based model simulation solutions on 8 real-world published SBML models retrieved from BioModels, **b** comparison of 3 MATLAB-based modeling solutions on 16 real-world models for which no SBML exist. They come from published literature retrieved from Pubmed (“PAPER”) or as benchmarks of the tool “MOCASSIN”, from “MATLAB” samples, from the Lysosomal Storage Disorders family of models (“LSD”), or in-depth “Case Study” discussed in the text ^39–46^.

We used 24 real-world projects to demonstrate QSPcc’s ability to translate a wide set of different models and algorithms and to improve their time performance. We extensively searched the literature using databases such as PubMed and Google Scholar for modeling papers describing a MATLAB implementation. From the results we identified, we considered only those satisfying these criteria: (1) the source was available as a set of MATLAB scripts, (2) it was of a significant dimension, (3) the code ran as-is on MATLAB. In Supplementary Table 1, we provide the comprehensive list of 62 test cases, including the 24 real-world projects, with running times and performance gains, including additional benchmarks. QSPcc automatically generated equivalent C code for every test. Results involving numerical integrations may contain negligible differences in the order of, or smaller than, the relative and absolute tolerances. This happens because MATLAB and C use different underlying algorithms and temporal adaptivity strategies to perform the integration (see Supplementary Tables 4 and 6). In Fig. 1a, we summarize the solutions available to execute standardized SBML representations and the performance gain of 8 mathematical models. In Fig. 1b, we summarize the solutions available to execute MATLAB-only model representations and the performance gain of 16 real-world mathematical models. These models cannot be handled by SBML solutions, while the topology optimization^10^ works only in QSPcc, other than MATLAB. The figures show the speedup, i.e., how many times each solution simulates faster than MATLAB, used as baseline (in gray).

It is worth noting that SBML representations can be easily converted to MATLAB due to their standardized form, while the vice-versa is not true in general. Further, the speed-ups depend mainly on the integrator used and the modeled biological reactions, while in general MATLAB code speed-ups can depend heavily on the model implementation. Table 1 summarizes the biological models of considerable size and relevance that have been directly developed by the authors using QSPcc.

**Table 1:** **Models of significant biological complexity and clinical relevance the authors developed leveraging the QSPcc compiler**.

	Model						
Metric	ASMD	Fabry	Gaucher	LSD platform	Tuberculosis (GranSim)	GBA-PD	Capuani2015
No. of equations	54	154	82	312	13 ODEs + rule-based interaction of cells	105	212
No. of parameters	117	234	83	506	44	150	16
Matlab time (s)	21.5	1168.2	74.9	25.7	166.2	1.6	12.5
QSPcc time (s)	1.41	3.1	2.5	0.2	68.3	0.003	0.2
Speedup	15x	377x	30x	128x	2.43x	533x	63x

Typical real-world complexity and challenges lie in the number of simulated equations, parameters, the overall stiffness of the system and the complexity in the code to meet the biological objectives. The EGFR Early Activation Model published in Capuani^38^ did not benefit of QSPcc. However, currently ongoing extensions do. For public reproducibility, the reported benchmark refers to the BioModels’ public version.

**Fig. 2:**
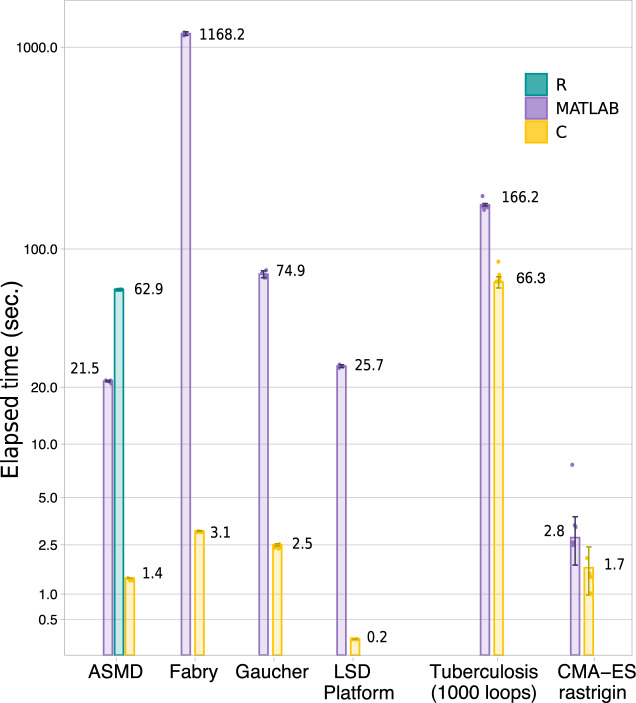
Runtime analysis of the case studies and lysosomal storage disorder models (LSD). Median running times, with the corresponding median absolute deviation (MAD) of the original MATLAB implementations, and the QSPcc-generated C and R benchmark models. Each model was run 5 times. From left to right. The three clinical lysosomal disease models, benefitting of 15x, 377x, and 30x speed-ups each, that we integrated in the fourth LSD platform^11^, which ran 128 times faster in the intensive model optimization phase. The R simulation is consistently slower than MATLAB. It is provided only for ASMD, one of the three representative in-dept case studies part of the LSD platform, as a demonstration of the compiler’s ability to work with multiple languages. On the right, the two in-depth case studies described in the text for Tuberculosis agent-based modeling and CMA-Es evolutionary optimization algorithm.

Each model was executed 5 times. The bar chart of Fig. 2 reports the median value and the MAD for each model. We observe that the C translation significantly improves the execution time in all the presented models. The R example of ASMD, provided as a demonstration of the compiler’s ability to work with multiple languages, runs much slower than the corresponding MATLAB case. This could be because MATLAB parallelize several operations automatically, while the R core functionality is intrinsically single-threaded. A full-stack R support can be openly pursued by the research community.

In the next section, we introduce the clinical lysosomal storage disorders research project^11^ where QSPcc was conceived, developed, and successfully applied. We then present in depth three use-cases from published literature. (1) Acid Sphingomyelinase Deficiency (ASMD)^12^, a deterministic QSP model based on a system of ODEs; (2) The Spatiotemporal development of granulomas in tuberculosis (GranSim)^13^, a hybrid Agent-Based/ODE framework; and (3) Covariance Matrix Adaptation—Evolution strategy (CMA-ES)^14–16^ a state-of-the-art evolutionary algorithm for the minimization of a target function used in optimization problems and model calibration. A visual overview of the test cases is presented in Fig. 2. For these complex models, a SBML counterpart is not available.

### Lysosomal storage disorders models

We developed the QSPcc compiler to enable an ambitious research project focused on creating an integrated computational platform to support research and therapeutic development for the sphingolipidoses^11^. The QSP platform was created incorporating three different large models of ASMD (described in the next section)^17^, Gaucher disease type 1^18^ and Fabry disease. Each model in turn had been developed incorporating and describing clinical trial and experimental data from approved and in-development drugs, biomarkers dynamics, metabolic and transport mechanisms. The integrated platform models organ-level clinical manifestations, genotype-phenotype relations, molecular metabolisms and drugs’ effects. As a counterpart for this level of details, the integrated platform has more than 300 equations and 500 parameters, resulting in a long simulation runtime in MATLAB. The use of QSPcc for the simulation provided a significant speedup (more than 100x, see Fig. 2) allowing the smooth progress of the research project.

In addition, QSPcc has also been successfully applied to support the development of an unsupervised stratification methodology^19^, which is also tested on the Gaucher disease^18^. The algorithm is based on a mathematical model description of a disease of interest and involves a global sensitivity analysis and multiple model calibration steps, requiring generally hundreds of thousands of model simulations. Thus, the computational effort of the method is strictly connected with the simulation runtime of the model. In the clinical test case described in the paper, the use of QSPcc allowed the authors to seamlessly employ a custom MEX function to simulate a QSP model of Gaucher disease type I^18^ while keeping the rest of the methodology in the MATLAB language. The MEX function halved the simulation runtime of the model, thus reducing the execution of the complete methodology from nearly one month to twelve days. More numerical details about the computational efforts can be found in the Supplementary Material of^19^.

### Acid sphingomyelinase deficiency

The development of QSPcc was initially aimed at enabling QSP model simulations in the LSD platform made of ASMD, Gaucher, and Fabry sub-models. Here, we analyze the ASMD model^17^, a recently published QSP framework describing non-neurological Acid Sphingomyelinase Deficiency (ASMD)^12,17,20^. ASMD is a rare lysosomal storage disorder caused by the reduced functional enzyme *acid sphingomyelinase*, leading to accumulation of *sphingomyelin* in multiple tissues resulting in clinical manifestations such as organomegaly and decrease in lung function. ASMD is a serious and potentially fatal disease. In^17^, the ASMD model is presented in detail and tested on different preclinical and clinical studies, including two human studies. Olipudase alfa (recombinant human *acid sphingomyelinase*, rhASM) is an enzyme replacement therapy under development for the treatment of the non-neurological manifestations of ASMD. The replacement of the deficient enzyme can clear the accumulated substrate and alleviate the symptoms of the disease. To model the action of rhASM, a system of 52 ODEs was implemented in MATLAB. QSPcc translated the ASMD model in C and R. To compare the results, we performed time-course simulations and collected timeseries of 200,000 steps for each variable of the ODE system. Then, we computed the relative error between MATLAB and R, and MATLAB and C, averaged over all the 52 variables at each time steps of the integration. We computed the relative error using the following formula.

$$\frac{{{{{{{\rm{abs}}}}}}}\left(A-B\right)}{{10}^{-6}+{{\min }}\left({{{{{{\rm{abs}}}}}}}\left(A\right),{{{{{{\rm{abs}}}}}}}\left(B\right)\right)}$$

where *A* and *B* are values of each variable computed by the source or target code, abs() is the absolute value and min the minimum between two values. The expression 10^−6^ at the denominator avoids the explosion of the relative error when the values are extremely small and does not affect the computation since it is significantly smaller than the relative tolerance, which was set to 10^−3^.

**Fig. 3:**
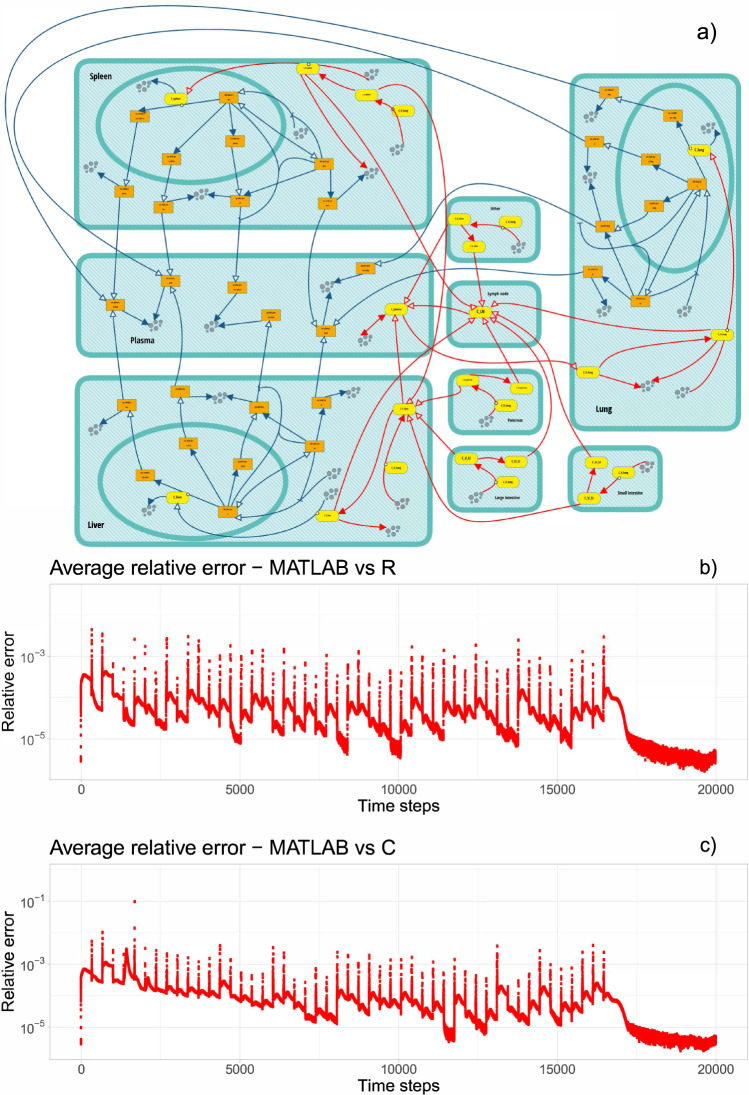
Model diagram of the acid sphingomyelinase deficiency (ASMD) with QSPcc model translation simulation accuracy. ASMD is a rare lysosomal storage disorder, here modeled treated with the investigational olipudase alfa enzyme replacement therapy. **a** ASMD model represented as a biological pathway in the bStyle environment^21^. **b** Relative error for each time step of integration, averaged over all variables of the target R model compared to the source MATLAB model. **c** Relative error for each time step of integration, averaged over all variables of the target C model compared to the source MATLAB model.

The comparison of the results and a graphical representation of the model, designed with the executable Visual QSP modeling platform bStyle^21^, are illustrated in Fig. 3. By default, the MATLAB ODE solver has a relative precision of 10^−3^, used also for the other modeling languages. The results of the translated models are in good agreement with the original results.

### Spatiotemporal development of granuloma in Tuberculosis

QSPcc can be applied beyond pure ODE-based models. For instance, GranSim^13^ is a hybrid Agent-Based/ODE model that describes the spatiotemporal development of granulomas. The model describes the growth of intracellular and extracellular Mycobacterium tuberculosis, and the containment efforts of several immune cell types within the broad categories of *macrophages* and *T cells*. The model was originally published in 2013, and subsequently several extensions (*i.e*., including other cell types, incorporating treatment effects, etc.) have been published. The model itself has been distributed as an executable, not in code. Supplementary documents describing the ABM rules and ODE structure were used to implement GranSim in the MATLAB environment^13^. The MATLAB implementation was then translated to C using QSPcc. For each agent of the model, the system modeled in this example consists of 13 ODEs describing the production of *TNF-α* and *IL-10* cytokines, the binding of ligand-receptor, and the release into the extracellular space. Each agent follows then a set of rules describing the movement on the simulated grid.

**Fig. 4:**
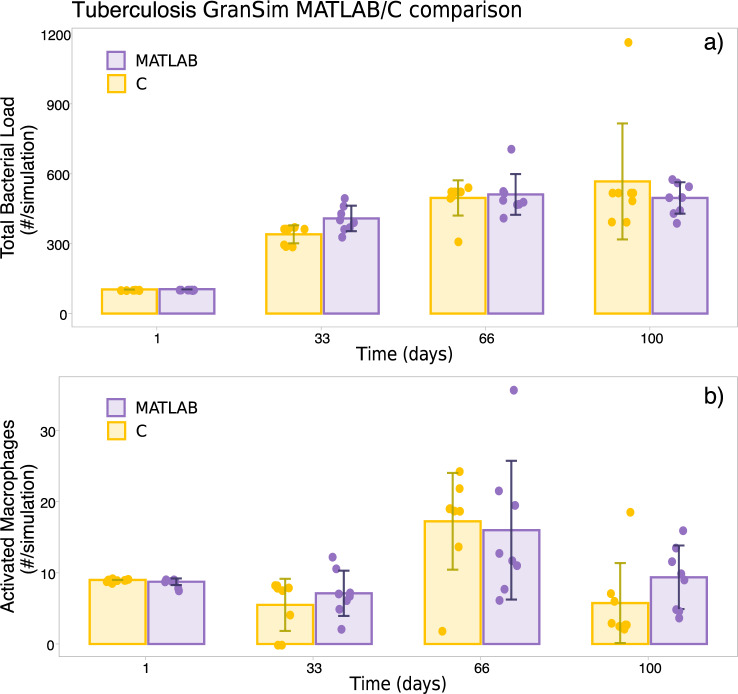
Correctness of the tuberculosis GranSim model translation. The error bars represent the standard deviation of the average over five simulation runs. **a** Total bacterial load and (**b**) number of activated macrophages at selected timepoints compared for MATLAB and QSPcc-generated C code.

Since GranSim is partially based on random moves, we simulated eight times both the original implementation and C translation and collected the statistics of two variables of the model (Total Bacterial Load and the number of Activated Macrophages) at several time steps, following the example of Fig. 4 in^13^. As shown in Fig. 4, the statistics show agreement between the two versions of the model.

### Covariance matrix adaptation—evolution strategy (CMA-ES), a non-linear, non-Newton evolutionary optimization algorithm

Another example that shows how QSPcc can be applied beyond the field of modeling and systems biology is the translation of the popular, state-of-the-art, CMA-ES algorithm, widely used in model parameters optimization. CMA-ES^14,15^ is an evolutionary algorithm for non-linear, non-convex black-box optimization problems. In contrast to quasi-Newton methods, CMA-ES does not use or approximate gradients and does not even require their existence. This makes the method feasible on non-smooth and even non-continuous problems, as well as on multimodal and/or noisy problems. It turns out to be a particularly reliable and highly competitive evolutionary algorithm for local optimization^14^ and for global optimization^15,16^. Even if C implementations of CMA-ES already exist (https://github.com/CMA-ES/c-cmaes), they are implemented from scratch. Assuming to take the MATLAB implementation as a reference, a change in the original algorithm will make them outdated and will require a manual intervention to update the code. With QSPcc, we were able to automatically translate the CMA-ES algorithm directly from its MATLAB implementation, meaning that any manual update of the MATLAB algorithm can be simply followed by re-running QSPcc to automatically obtain the updated C translation.

**Fig. 5:**
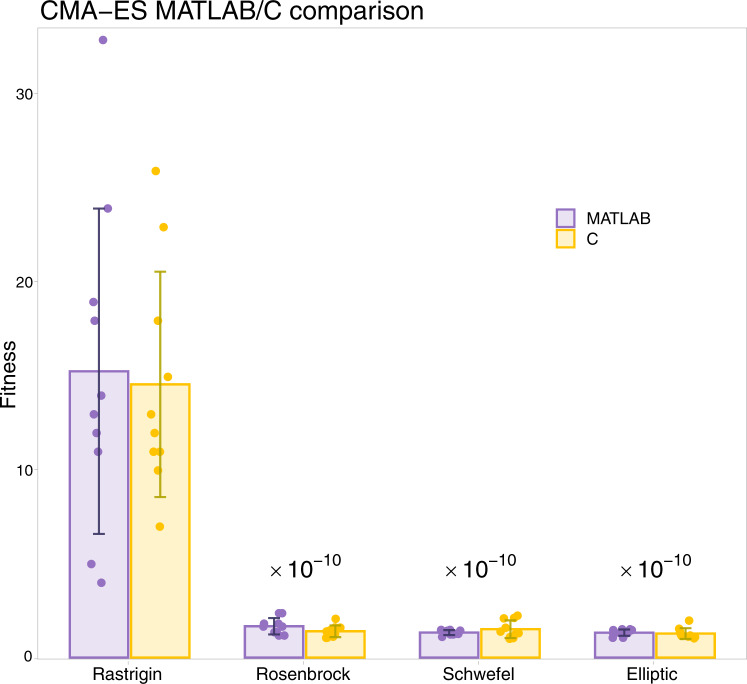
Correctness of the evolutionary optimization CMA-ES algorithm translation. Average and standard deviation over ten executions of the CMA-ES fitness algorithm for some typical benchmark functions ran in MATLAB and QSPcc-generated C code. The times of the three benchmark functions of the right are multiplied by 10^−10^ to keep them all on the same scale.

Tests of the CMA-ES translation were executed on some popular benchmark functions (Rastrigin, Rosenbrock, Schwefel, and elliptic functions)^22^. We ran the algorithm ten times and compared the average fitness (the minimum of the target function) between the MATLAB and the C results in Fig. 5. Again, the dynamics of the translated version of the model agrees with one of the original models.

### Comparison with other tools

Sixteen real-world, MATLAB-only models are listed in Fig. 1b, in which we compare the runtime improvement of QSPcc C translation with MATLAB and MATLAB Coder (https://www.mathworks.com/products/matlab-coder.html). We also tested, and excluded from the figure, AMICI^23^, CVOde Wrapper^24^ and matlab2cpp^25^ because they require the MATLAB code to be entirely rewritten to fit their own format/coding style. We tested Sbaddon^26^ in a previous version with similar outcomes, but we were unable to install it on a recent MATLAB installation. The compiler-based approach in QSPcc allows to read the normal MATLAB syntax without changes.

Further, considering the interoperability value, we compared eight manually curated SBML model representations with a wide range of alternative solutions: MATLAB, MATLAB Coder, AMICI, libRoadRunner and COPASI. Our results are depicted in Fig. 1a. The MATLAB code for the models was downloaded from the BioModels web portal (https://www.ebi.ac.uk/biomodels/).

MATLAB is a comprehensive framework and a programming language that expresses matrix and array mathematics easily. It is currently a de-facto standard in the field. It provides a C interface named C/MEX (https://www.mathworks.com/help/matlab/ref/mex.html), a C dialect that can be compiled and run within MATLAB to speed up critical computations. The drawback of C/MEX is that the user should be able to hand-write correct C code and correctly interface it with the MATLAB libraries.

Another solution is MATLAB Coder (https://www.mathworks.com/products/matlab-coder.html), a MATLAB module that can automatically translate the MATLAB language and some built-in MATLAB functions to C/MEX code as well as standalone C++ code. The main drawback is that some common functions (such as ODE solvers for stiff problems), are still not supported in standalone mode, while the alternatives (such as ode23 or ode45) are too slow in the LSD diseases we were modeling, as well as in the benchmarks, see Fig. 1. Further, it does not support scripts, structured projects (addpath is not supported), does not handle comparisons between scalars and matrices, does not allow to write output directly in standalone mode (disp is ignored, writematrix, load, save are unsupported), requires variables to (1) be always initialized in the main function block before use (2) that matrices should not be resized during execution (this can be circumvented by adding specific code for every dynamic variable). To successfully translate LSD models with Coder, we had to heavily change the MATLAB code, and it took an expert engineer who knew the models an average of 3 hours per model with a peak of 6 hours trying to translate GranSim.

SBaddon is an extension package to the Systems Biology Toolbox for MATLAB, focusing on parameter estimation problems. It allows to speedup integration by exploiting the Sundials library, but it is discontinued since 2006, and we were not able to install it on a recent MATLAB release.

Other solutions, such as CVode wrapper, AMICI and matlab2cpp provide means to semi-automatically translate from MATLAB to C/MEX but requires ad-hoc engineering of the MATLAB code to adhere to a specific code format or to manually complete the translation, thus preventing translation of large, complex or already-existing models. Matlab2cpp is a semi-automatic translation tool, where user should manually fill all data types required by C++ code.

Visual tools^21,27^ allows to draw and manipulate models represented as a network of reactions. As such they can be exported to, and/or imported from, SBML or any other reaction-based model representation. The drawback is that not all models can be represented as a network of reactions (for example, the use-case GranSim is not even an ODE model). There are systems biology solutions that allow us to convert from MATLAB to SBML^28^, but in general, code cannot be automatically understood and represented as a network of reactions other than in a reduced number of cases^28^.

Finally, some solutions, such as libRoadRunner, AMICI and COPASI, directly execute a reaction-based representation, such as SBML, and does not require changes to the code to run. We benchmarked QSPcc against manually curated models represented in SBML by downloading their MATLAB equivalent from the BioModels website.

The results summarized Fig. 1 demonstrate the general applicability of QSPcc even on standardized SBML representations, and at the same time shows a significant speedup compared not only to MATLAB, but also to any other alternative solution.

From the results, we can draw three main conclusions: (1) QSPcc-generated C always outperforms MATLAB, and the other solutions, in most of the cases and (2) Existing solutions are able to handle fewer cases compared to QSPcc (3) Existing solutions require manually tweaking of the original code, while QSPcc is able to work out of the box. For these reasons, QSPcc is better suited for large, already existing, MATLAB models, where re-implementing the model in another language or heavily adapting the existing one is not desirable or feasible.

Another goal of QSPcc is to improve the time performance of model simulation and in the Supplementary Table 1 file we report the results on 62 test models. QSPcc can leverage different ODE solvers and libraries to speedup the computation, including the C ODE solver library sundials from version 2.7 to 5. Since QSPcc is being actively developed, it defaults to the latest sundials version^29^. Highly efficient math library MKL^30^ version 2020 can be also used to speedup matrix-based operations.

## Discussion

In several research fields, including natural science and QSP, the runtime of modeling and simulation is becoming a crucial issue. Mathematical modeling is the art of simplifying a complex phenomenon in terms of a mathematical description that is both easily understood by humans and computationally tractable by machines. In recent years, however, models have increased in complexity to capture the phenomenological realism required to describe the dynamical properties of the modeled biology. The execution time may become a bottleneck for more detailed and complex mathematical models, which demand more computational resources, especially using higher-level modeling languages. To mitigate these issues we were facing, to enable the simulation of previously intractable problems by speeding up the simulation, and allowing seamless translation between modeling languages, we developed and present QSPcc, a new compiler-based translation tool.

QSPcc translates models from a source language, in our test cases MATLAB, to another programming language, in our test cases C or R. We demonstrated with several examples that the results of the translated models are as accurate as the source, whereas the running time improvement often enables the execution of previously intractable problems. In addition, we compared and benchmarked QSPcc against the currently available solutions proving QSPcc to be significantly faster and comprehensive than other state-of-the-art methods. Moreover, QSPcc allows modelers to quickly translate part of their MATLAB code into efficient C/MEX that can be seamlessly included in other MATLAB projects.

QSPcc works out of the box on the source code and does not require code manipulation or a specific syntax prior to the translation. QSPcc can automatically translate a large set of mathematical expressions, such as linear algebra operators and mathematical functions. Furthermore, even complex functions such as *load*, *setdiff*, *union*, *interp1* in MATLAB are seamlessly translated to equivalent target language code (the full list of constructs currently supported is reported in Supplementary Table 3). On the contrary, many existing solutions demand to manually tweak the source code, and thus, to maintain two versions of the same model, one for MATLAB and one for the translator. The far greater number of programs that QSPcc translates with respect to competitors demonstrates that our tool is more flexible and general-purpose than other tools available. QSPcc runs on all modern Operating Systems thanks to our pre-configured Docker container^31^. In this way, it does not require strong programming skills to optimize arbitrary models execution nor system administrators’ assistance to install Java, C compilers and configure C libraries.

QSPcc is modular as it allows any developer to easily extend its functionalities in many ways. For example, new functions of the source language can be easily included by either providing the code for the function in the source language or by providing a translation in the target language. It is also possible to add new languages previously unsupported such as Julia^32^ or Python, either as a source language or as a translation target. Analogously, the implementation of existing languages could be further extended to add new language constructs or increase time efficiency. As an example, we developed the R language support necessary to translate the ASMD model in R. The QSPcc R backend already correctly executes 35 out of the 62 test models and is provided as a demonstration of the compiler’s flexibility and an occasion for further extension. See the QSPcc documentation (https://github.com/cosbi-research/QSPcc) for further instructions.

Furthermore, QSPcc is open-source under the BSD3-Clause license. Currently, we support the open-source languages C and R, thus allowing users to choose between licensed software and open-source applications to run their simulation or algorithms. Commercially licensed software such as MATLAB ease development and debugging but they require a MATLAB license to run the model. On the other hand, QSPcc allows researchers to execute MATLAB code in a non-MATLAB environment, and integrate multiple languages (*e.x*. R and MATLAB) in a single translated executable such as C or MEX. Refer to Supplementary Tables 3, 4, 5 and 6 for the mappings among them.

Among the complete list of benchmarked cases (see Supplementary Table 1), we have selected a MATLAB implementation of a computationally intensive engineering algorithm named topology optimization, refer to^10^ for the details. The QSPcc-generated C translation produced the correct result, but the running time execution of the C code resulted in only a 5% improvement over the original MATLAB model (2.8 secs for C versus 2.93 secs in MATLAB) even when using the hardware-accelerated Intel MKL library sparse matrices and related operations implementation. The exact motivation is to be investigated further and is most probably related to either a suboptimal C implementation of matrix operations or to fast built-in matrix operations in MATLAB.

As a future improvement, we are planning to optimize even further the C code generation, to expand the number of supported constructs, and to gradually expand the coverage and efficiency of the R support as well.

Finally, QSPcc increases the portability of the models and algorithms by easing the transition between languages. An algorithm in MATLAB could be made accessible to researchers familiar with a different scientific language such as R, C or provided that a corresponding language module was developed, Octave, Python or Julia. QSPcc generates code providing a side-by-side comparison of languages, helping people learn and understand less familiar languages used by the collaborators. After the translation, the model can be extended with libraries belonging to the target language that are not present in the source language.

We sincerely hope to support the wider community’s research efforts until new disruptive computational platforms become a readily accessible reality.

## Materials and Methods

**Fig. 6:**
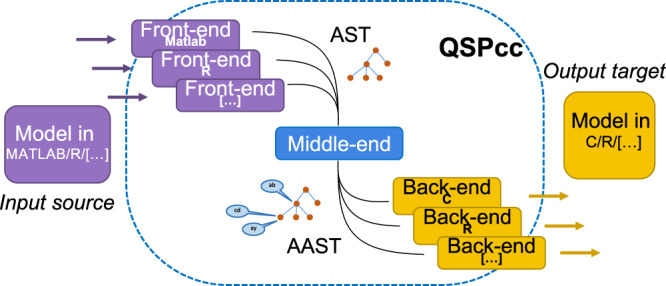
High-level QSPcc compiler organization. The sequence of transformations the input source code undergoes during the translation is rooted in formal methods in computer science supporting the biological applications described here. A bird eye outlook shows how a dynamical phenomenon, as the LSD family of models, written in a source programming language is transformed in a Tree (AST). This is further semantically annotated becoming a formal data structure (AAST) allowing to understand the meaning of the original model to guide the construction of a more efficient representation in a target language.

QSPcc is a language translator tool, empowering researchers with state-of-the-art compiler technologies. It implements a source-to-source compiler able to build an abstract representation of the input language and to generate the target language output from the abstract representation itself. The tool can be logically divided into three main blocks organized in a workflow as illustrated in Fig. 6, namely (1) Front-end language analysis (2) Middle-end Abstract Syntax Tree enrichment, (3) Backend target language generation. Here, we describe in detail the operations performed by each block of the QSPcc pipeline.

### Front-end

The aim of the front-end block is to perform lexical syntax analyses. At the beginning, the front-end splits the input files in *tokens* that are relevant for the input language. The front-end is able to recognize variable identifiers, assignments and keywords corresponding to reserved statements of the source language. Then, the front-end performs a syntax analysis. The token list is organized in a tree structure called *Abstract Syntax Tree* (AST)^33^. The process of organizing the tokens is called *parsing*. Parsing follows the rules defined in a *formal grammar* that defines the syntactical structure of the input language. Every front-end builds a Directed Acyclic Graph of ASTs called *Program* to be used by the middle-end for the subsequent analysis phase.

For the MATLAB front-end, we wrote the grammar with the parser generator ANTLR version 3^34^ to perform the tokenization and the AST tree generation.

### Middle-end

The middle-end is the core part of QSPcc that performs the semantic analysis on the AST. The middle-end takes as input the un-annotated AST built by the front-end and returns the same AST enhanced with type information for the different nodes of the tree. We refer to this enhanced version as Annotated AST (AAST).

The annotation process includes three main sub-operations that together generates the AAST. (1) Type inference, namely the automatic detection of the variable types, the identification of the function parameters and output values. (2) Type checking, namely checking the compatibility between the variables and the operators they are applied to. For instance, the MATLAB quotient ‘/’ between two 1-dimensional vectors is an undefined operation, as the point-wise quotient is already defined by ‘./’. (3) Environment checking, namely checking if used variables and functions belong to their scope. For instance, a function g defined inside a function f, will be available for use only inside the function f, any call to g outside f will be undefined.

The middle-end acts as a full multi-pass compiler that collects type information for variables and functions refining type and scope information at every subsequent step until every variable and function is fully defined.

In this way, eventually, every function and every variable will get its type, and in some cases even more than one possible type since limited polymorphism is allowed. The iteration stops when all the variables and all the functions in the tree are fully defined. This mapping is independent on the order the variables are defined given that every variable is defined before use. The AAST built in this way is equivalent to the Intermediate Representation (IR)^33^ of the program that many compilers, such as the gcc C compiler, write as an iterative sequence of low-level commands. The advantage of having a tree shaped AAST versus an IR is the readability and ease of debugging and visualization.

The AAST produced by the middle-end can be either passed along the translation chain to the configured backend or rendered in SVG using the **dot** visualization tool^35^ that belongs to the graphviz library bundled within QSPcc. This allows developers to debug easily the middle-end behavior, and users to understand what type is assigned to variables in the source code.

### Backend

The backend translates the AAST to code in the target language. Thanks to a careful translation, the input Program does not need to be in *static single assignment* form like other MATLAB compilers require^36^, and also limited type polymorphism is allowed (Ex. matrices can be used as scalars and vice-versa). This flexibility allows modelers to focus on developing the model rather than on translation issues.

C backend. C backend generates C code, compatible with all the main C compilers such as gcc (GNU C Compiler), clang (llvm C compiler), icc (intel C compiler) and pgc++ (NVIDIA C compiler), and with C/MEX that can be used as a building block in a MATLAB project. Since C is more verbose than MATLAB, comments reporting the source line are copied above each translated statement, making it easy to inspect and modify the output. Furthermore, the generated C code is also automatically parallelized on CPU with openMPI^37^ and hardware-optimized implementations can be used whenever possible with the Intel Math Kernel Libraries (MKL). ODE models are simulated with the Sundials solver^29^. During the simulation, QSPcc C translation can automatically recognize out-of-bound variables (such as NaN, Infinity ecc) and report to the user the current time step and the variable(s) out of bounds.

R backend. Another backend included in QSPcc is the R backend. In the current implementation, this module is able to translate a good subset of MATLAB models to R. However, this module was developed as an example and has not been brought to the level of the C backend.

### Reporting summary

Further information on research design is available in the Nature Research Reporting Summary linked to this article.

## Supplementary information

Supplementary Information

Reporting Summary

## Data Availability

All data supporting the findings in this study are available as mathematical models either in the public GitHub repository or in the discussed referenced articles. The source data underlying all figures and tables are available at https://www.cosbi.eu/fx/298319232/qspcc_source_data.zip.

## Code availability

QSPcc is publicly available under the BSD-3 license at https://github.com/cosbi-research/QSPcc. 10.5281/zenodo.5036945.
