# Supplementary material for: QSPcc reduces bottlenecks in computational model simulations
Source: Commun Biol. 2021 Sep 1;4:1022. doi: 10.1038/s42003-021-02553-9 (PMC8410852; doi:10.1038/s42003-021-02553-9)
Supplement: Supplementary file 1 — Supplementary Information [file 42003_2021_2553_MOESM1_ESM.pdf]

Supplementary Information

QSPcc reduces bottlenecks in computational model simulations

Danilo Tomasoni[1], Alessio Paris[1], Stefano Giampiccolo[1], Federico Reali[1], Giulia Simoni[1], Luca Marchetti[1], Chanchala Kaddi[2,], Susana Zaph[2], Corrado Priami[1,†], Karim Azer[2,+], Rosario Lombardo[1,*]

[1] Fondazione the Microsoft Research - University of Trento Centre for Computational and Systems Biology, Rovereto, Italy
[2] Translational Disease Modelling, DDS, Sanofi, Bridgewater, NJ, USA

† present address: Department of Computer Science,University of Pisa, Pisa, Italy
+ present address: Axcella Health, Cambridge, MA, USA

* Corresponding author

Rosario Lombardo

E-mail: lombardo@cosbi.eu

1 PROPRIETARY TEST MODELS 3

1.1 MODEL OF GAUCHER DISEASE TYPE 1 3

1.2 MODEL OF FABRY DISEASE 3

1.3 MODEL OF LYSOSOMAL STORAGE DISEASES 3

1.4 MODEL OF GBA-PD 4

2 PUBLICLY AVAILABLE TEST MODELS 4

3 ALL BENCHMARKED MODELS 6

4 USAGE 8

5 EXTENDING QSPCC 9

6 SUPPORTED EXPRESSIONS 10

6.1 NUMERICAL ODE SOLVER METHODS 20

6.2 ODE NUMERICAL SOLVER PARAMETERS 24

1 PROPRIETARY TEST MODELS

QSPcc was originally developed and successfully used to improve or enable the simulation of some proprietary models. Later the coverage was extended to support a wider set of constructs. The following paragraphs contain a short description relative to the proprietary QSP models.

1.1 MODEL OF GAUCHER DISEASE TYPE 1

Gaucher disease type 1 (GD1) is a lysosomal storage disease characterized by the lack of the Glucocerebrosidase (GBA) enzyme, which leads to the accumulation of glucosylceramide in several tissues such as liver and spleen. The GD1 model is a QSP description of the disease and of the effect of two types of therapies, the enzyme replacement therapy (ERT) and the substrate reduction therapy (SRT). The GD1 QSP model provides a mechanistic computational platform for predicting treatment response via different modalities within a heterogeneous GD1 patient population. The model consists of 82 equations and 83 parameters and has been published in 2020.

In our tests, the QSPcc translation of GD1 in C is 30 times faster than the MATLAB version.

1.2 MODEL OF FABRY DISEASE

Fabry disease is a lysosomal storage disease characterized by the lack of the alpha-galactosidase A enzyme, which leads to the accumulation of globotriaosyceramide in tissues such as the kidneys and heart. Venglustat is a small molecule that is under clinical development for the treatment of multiple glycosphingolipids dysfunction, including Fabry disease. To support the development of Venglustat for Fabry disease, a quantitative systems pharmacology (QSP) model was developed to predict the effects of Venglustat in different Fabry patient cohorts across different dosage regimens.

The model consists of 154 equations and 234 parameters and is currently under review for publication.

In our test, the QSPcc translation of Fabry in C is 377 times faster than the MATLAB version.

1.3 MODEL OF LYSOSOMAL STORAGE DISEASES

LSD is an integrated QSP platform to support research and therapeutic development for the sphingolipidoses. The platform provides a single model able to describe the dynamics of acid sphingomyelinase deficiency (ASMD, Gaucher disease type 1 (GD1), and Fabry disease. In addition, the platform supports the simulation of different treatments with the aim of comparing the efficacy and explore the drug repurposing of disease specific treatments in the contest of the other diseases.

The model consists of 312 equations and 506 parameters and is currently under review for publication.

In our test, the QSPcc translation of LSD in C is 128 times faster than the MATLAB version.

1.4 MODEL OF GBA-PD

The mutations of the GBA1 gene, whose autosomal mutations are responsible for Gaucher disease, are among the strongest genetic risk factors for the development of Parkinson’s disease (PD). The GBA-PD model is a multi-level model that investigates the interaction between the GBA mutations, the glycosphingolipids metabolism and the development of PD.

The model consists of 105 equations and 150 parameters and is currently under review for publication.

In our test, the QSPcc translation of GBAPD in C is 533 times faster than the MATLAB version.

2 PUBLICLY AVAILABLE TEST MODELS

In this section, we describe the other MATLAB test models we used to validate the QSPcc time performance improvements.

MOCASSIN models were taken from the third-party MOCASSIN project and they represent small real world ODE simulation problems on which the MOCASSIN tool can be evaluated. One of them, the “distefano” model was broken. Despite this, MATLAB gives no warnings or errors about the simulation outcome, that results in a matrix of NaN. QSPcc C translation instead nicely handles the case of unworkable mathematical operations stopping the simulation and displaying a clear error message stating the variable that became NaN.

The *remaining test models* come from different sources:

- **Averaging filter** is a MATLAB example program taken from the MATLAB website, and computes a low-pass filter by implementing an averaging window https://www.mathworks.com/matlabcentral/fileexchange/40174-average-filter
- **Matlab2cpp** is the test program used by the matlab2cpp tool as an example https://github.com/jonathf/matlab2cpp#an-illustrating-example
- **Parametrized Lorenz** is an example used by the Julia programming platform adapted to run on MATLAB. It computes the Lorenz system of ODE https://docs.juliadiffeq.org/latest/tutorials/ode_example.html#Defining-Parameterized-Functions-1

- **Cartesian to geodetic** is the MATLAB code of the paper "An alternative algebraic algorithm to transform Cartesian to geodetic coordinates" https://doi.org/10.1007/s00190-005-0487-5
- **FIRM** is the MATLAB code of the system biology ODE model published in "The development of a fully-integrated immune response model (FIRM) simulator of the immune response through integration of multiple subset models." https://www.ncbi.nlm.nih.gov/pubmed/24074340
- **Topology Optimization** is an efficient 88 line MATLAB code for topology optimization. The authors refers to it as a practical instrument that may help to ease the learning curve for those entering the field of topology optimization. http://doi.org/10.1007/s00158-010-0594-7
- **A neural network model of Parkinson's disease bradykinesia** a comprehensive network model to study how patterns of DA depletion at key cellular sites in the basal ganglia, cortex and spinal cord contribute to disordered neuronal and spinal cord activity and other PD symptoms https://doi.org/10.1016/j.neunet.2005.08.016
- **A Computational Study of Stimulus Driven Epileptic Seizure Abatement** study of a neural population model of spike-wave seizures that allows the reconstruction of the basin of attraction of the background activity as a four dimensional geometric object http://doi.org/10.1371/journal.pone.0114316
- **Winter2017:** Mathematical analysis of the influence of brain metabolism on the BOLD signal in Alzheimer's disease http://identifiers.org/doi/10.1177/0271678X17693024
- **Pathak2013**: Modeling of the MAPK machinery activation in response to various abiotic and biotic stresses in plants by a system biology approach http://identifiers.org/pubmed/23847397
- **Giordano2020**: Modelling the COVID-19 epidemic and implementation of population-wide interventions in Italy. http://identifiers.org/pubmed/32322102
- **Bertram2004:** Calcium and glycolysis mediate multiple bursting modes in pancreatic islets. http://identifiers.org/pubmed/15347584
- **Brännmark2013 :** The paper describes insulin signaling in human adipocytes under normal and diabetic http://identifiers.org/pubmed/23400783
- **Muraro2011:** The influence of cytokinin-auxin cross-regulation on cell-fate determination in Arabidopsis thaliana root development http://identifiers.org/pubmed/21640126
- **Capuani2015:** ODE model of EGFR phosphorylation and ubiquitination at the plasma membrane in HeLa cells. https://pubmed.ncbi.nlm.nih.gov/26264748/

The remaining *synthetic models* were built to test QSPcc functionality on single-function MATLAB models.

In Supplementary Table 1 we list the run times used to generate Figure 1 and Figure 2, and the corresponding number of times the C version is faster than the MATLAB version.

3 ALL BENCHMARKED MODELS

The tests were performed on a quad-core Linux box with the following hardware:

- Intel(R) Core(TM) i5 CPU cache: 8192 KB @ 3.20GHz
- 8 GB of DDR2 RAM (speed 1333 MHz).

The platforms used are:

1. MATLAB R2021a 64bit linux
2. R 3.4.3
3. gcc version 5.4.0 20160609

TEST MODEL	MATLAB	C	BOOST
CASE STUDIES			
ASMD	21.54	1.36	15.8x
GranSim	166.21	68.26	2.4x
CMA-ES	2.85	1.68	1.6x
**LYSOSOMAL STORAGE DISORDERS MODELS**			
Fabry	1168.16	3.07	380.5x
ASMD + Fabry + Gaucher	25.68	0.2	126.2x
Gaucher	74.9	2.45	30.5x
MOCASSIN			
Nudelman	0.1583	0.0000752	2105x
Sbmltoolbox	0.40296	0.0015696	256x
Hayot	0.27356	0.0000866	3158x
Handel	0.30312	0.0162	18.7x
OTHER TEST MODELS			
Averaging filter	0.00616	0.0009864	6.2x
matlab2cpp	0.0002056	0.0000008	257x
Parametrized lorenz	0.24278	0.0000163	14894x
Cartesian to geodetic	0.0153	0.00000180	8500x

Topology Optimization on a 30x10 grid	0.4663	0.377	1.6x
Topology Optimization on a 60x20 grid	2.86518	2.76	1.03x
Parkinson's disease bradykinesia	1.17	0.13879	4.83x
Stimulus Driven Epileptic Seizure Abatement	0.52	0.000999	520.52x
**SBML TEST CASES**			
Capuani2015	12.52	0.17	71.8x
Winter2017	607.617	0.028	21700x
Pathak2013	0.976	0.028	34x
Giordano2020	0.6887	0.0138	49.8x
FIRM	0.45358	0.0086744	52.2x
Bertram2004	0.6734	0.016	42x
Brännmark2013	146.2317	0.0205	7130x
Muraro2011	58.4895	0.0319	1830x
SYNTHETIC TEST CASES			
Linear solve	0.0841	0.0000214	3929x
matrix_fun	0.0028	0.0000226	123x
anonymous_fun_env	0.2507	0.000251	998x
Fun_concatenation	0.0031	0.0000054	574x
fun_multiple_returns	0.0027	0.000009	300x
time	0.0214	0.0000398	537x
parfor	0.0015	0.000009	166x
for	0.0018	0.000009	200x
while	0.1173	0.000102	1150x
if	0.0018	0.000009	200x
matrix	0.0023	0.0000072	319x
ordinary_integration	0.0486	0.0001256	386x
odeset	0.0335	0.000087	385x
ODEWrap simple	0.2187	0.0002944	742x
integration_in_specified_times	0.5417	0.0000754	7184x
scalar	0.0013	0.0000092	141x
boolean	0.0027	0.0000104	259x
multipleassign	0.0058	0.000008	725x
transpose	0.001	0.0000076	131x
Concatenation	0.0177	0.0000226	783x
Nodim (without MKL)	0.285	35.3119	0,008x
Nodim (with MKL)	0.285	0.368	0,77x
slice	0.0112	0.0000882	126x
multiple_load	0.018	0.0002852	63x
csv_load_header	0.0382	0.00127	30x
subroutine	0.0106	0.000037	286x
reshape	0.0047	0.00005	94x
random	0.000515	0.000256	2x
ismember	0.0229	0.000052	440x

cumsum	0.0015	0.000044	34x
concatenation	0.0043	0.000036	119x
type_generalization	0.000485	0.000008	60x
isempty	0.0018	0.000231	7.7x

Supplementary Table 1 Run time and boost for the tested models and matlab programs.

4 USAGE

Check the latest documentation online at https://github.com/cosbi-research/QSPcc

QSPcc is a command line tool with the syntax

qspcc --from < LANG > --to <LANG> --source <PATH> --dest <PATH> [-i <PATH>] [target options]

The list of main arguments is specified in Supplementary Table 2 Base command line options and arguments supported by the tool.the following Supplementary Table 2.

Argument/option	Mandatory/ Optional	Description
-f / --from <LANG>	Mandatory	Specifies the source language. Supported language options for <LANG> are: “Matlab” (case-insensitive).
-t / --to <LANG>	Mandatory	Specifies the target language. Supported language options for <LANG> are: “R”, “C” (case-insensitive).
-s / --source <PATH>	Mandatory	The path to the main source file
-d / --dest <PATH>	Mandatory	The destination folder.
-i / --input <PATH>	Optional	The CSV the user is planning to use as input for the generated program. The headers will be recognized as global 1D matrix variables. Some optimization techniques in **Error! Reference source not found.** can be enabled only using this option.

Supplementary Table 2 Base command line options and arguments supported by the tool.

Further options are available based on the target language (-t option) for a complete list see README.md in the QSPcc repository.
All the files produced by the compiler are saved in the destination folder.
In addition to the generated files, the destination folder will contain some libraries and the files needed to compile the program (*Makefile* and *Makefile.mac*) on Linux and macOS platforms, respectively, if the target language is C. Provided that a C compiler (one among GCC, ICC, CLANG or PGC) is available, mandatory libraries

are installed and the Makefile library paths is adjusted to match the current system paths, you can compile the C code by executing (macOS example):

```
make –f Makefile.mac
```

Users can compile the program and thus generate the object files in the same folder. Once the program has been compiled, users can run it with the executable file corresponding to the main script.
In the R case, the program does not need to be compiled, so users have just to run the main R script.
On Linux machines, a system-wide installation is available running the command *install.*
After each translation execution, a detailed log of the execution with errors and warnings is included in the folder *logs* inside the folder containing the QSPcc executable file. An automatic mechanism stores older log files on a daily base.
QSPcc can also be shipped on request as a Docker container with all the dependencies pre-installed, see README.md for further instructions on how to use it also on Windows.

5 EXTENDING QSPCC

The QSPcc SDK allows to extend QSPcc with extra functions and source or target languages. It contains an in-house library in JAVA to easily work with AST and AAST. The SDK library can be used to expand existing front-ends and back-ends or to develop new ones corresponding to additional source and/or target languages.
Here we highlight briefly the main classes of the library:

1. An **AASTNode class**, that represents a node in an AAST tree. In terms of code, a node is the smallest code unit worth representing. For example, the symbol '*' will be an AASTNode node of type 'TIMES' in the AAST tree.
2. An **AAST class**, that represents a full AAST tree. In terms of code, an AAST is a compilation unit, that is a single file with all it's operations. For example, the file 'main.m' will be an AAST.
3. A **Program class**, that represents the complete program to be translated. In terms of code, a Program is a tree of AAST, where the root of the tree is the main script where the program will start the execution, and the leaf of the tree are the most inner user-defined functions called by the program.

Check the latest documentation online at https://github.com/cosbi-research/QSPcc/blob/master/SDK_DEVELOPERS_GUIDE.md

6 SUPPORTED EXPRESSIONS

In Supplementary Table 3,Supplementary Table 2 we report an extensive list of how MATLAB statements are translated to the corresponding target language expressions in R and C. If the translation of a statement is not supported by one of the target languages, this is indicated by the label “Not implemented”. Some C functions contain one or more question marks (for example *??Max(A)*). Running a real example, the question marks are replaced by characters according to the inferred type of input and/or output data.

MATLAB	R	C
K=[1, 2]	K=c(1,2)	*Matlab matrices and arrays are translated with the C-structs*<br><br><type><dimension_number>dMatrix<br><br>*Where*<br><br>struct<br><type><dimension_number>dMatrix<br>{<br>type* matrix;<br>int dim1;<br>….<br>Int dimN;<br>}<br><br>*For example, the matrix in the example will be translated with the following code.*<br><br>int1dMatrix K;<br>static int K_vals[2];<br>K.matrix = K_vals;<br>K.dim1 = 2;<br><br>K.matrix[0] = 1;<br>K.matrix[1] = 2;<br><br>*It is worth noticing that the value array is statically allocated just when the matrix*

		dimensions are known at compile time, otherwise a dynamic allocation will be performed.
K=[3; 4]	K = rbind(c(3), c(4))	int1dMatrix K;<br>static int K_vals[2];<br>K.matrix = K_vals;<br>K.dim1 = 2;<br><br>K.matrix[0] = 3;<br>K.matrix[1] = 4
K= [1, 2; 3, 4]	K= rbind(c(1,2),c(3,4))	static int K_vals[4];<br>K.matrix = K_vals;<br>K.dim1 = 2;<br>K.dim2 = 2;<br><br>int2dMatrix U ;<br>static int U_vals[4];<br>U.matrix = U_vals;<br>U.dim1 = 2;<br>U.dim2 = 2;<br><br>K.matrix[0] = 1; K.matrix[1] = 2;<br>K.matrix[2] = 3; K.matrix[3] = 4;<br>*/
K= [1, 2; 3, 4]<br>U=K’	U<- t(K)	transposeMatrix(U.matrix, K.matrix, K.dim1, K.dim2);<br><br>*where* transposeMatrix *is a function in the* matrixLib *library that iterating just once through the original matrix elements populates the target one.*
K(1,2)	K[1,2]	*Elements of matrix have to be accessed through the flattened array* .matrix*, the function* matrixAccess *calculates the flattened index corresponding to the n-dimensional access vector (in this case (1,2)).*

		The example will thus be translated with:<br><br>K.matrix[matrixAccess(num_dim_K =2, num_access_parameters=2, K.dim1 , K.dim2, 1, 2)];
K(1,:)	C<-K[1,]	In order to slice a matrix, the *?SliceMatrix* function (in the Matrix library) is used.<br><br>This function takes as input an array of integer triplets, standing for the slicing beginning, step and end on each dimension.<br><br>*For example the K(1, :) statement will be translated with following code*<br><br>int idxs0_slice_array[2][3] =<br>{<br>{ 1, 1, 1 },<br>{ 1, 1, 2 }<br>};<br><br>char idxs0_slice_array_type[2]<br>= {<br>0, 0<br>};<br><br>intSliceMatrix(c.matrix, K.matrix, 2, idxs0_slice_array, 2, idxs0_slice_array_type, 0, 2, 2);
K(1,end)	K[1,ncol(K)]	*A matrix access on the first row and last column is performed, if the matrix has two dimensions it will be translated with:*<br><br>K.matrix[matrixAccess(Num_K_dimensions, Num_access_parameter, K.dim1 , K.dim2, 1, K.dim2)];
K= [1, 2; 3, 4]<br>C = K(end,1)	K[nrow(K),1]	*A matrix access on the last row and first column is performed,*

		if the matrix has two dimensions it will be translated with:<br><br>K.matrix[matrixAccess(Num_K_dimensions, Num_access_parameter, K.dim1 , K.dim2, K.dim1, 1)];
K(end)	K[length(K)]	*A matrix access on the last row and last column is performed, if the matrix has two dimensions it will be translated with:*<br><br>K.matrix[matrixAccess(Num_K_dimensions, Num_access_parameter, K.dim1 , K.dim2, K.dim1, K.dim2)];
[t,y] = ode45(odefun,tspan,y0)	t_y=ode(…)<br>t=t_y[,1]<br>y=t_y[,2:ncol(t_y)]<br><br>*where ode(..) is described in the next chapter*	*Once solved the differential equation with the Sundials library (as specified in the following chapter), a double slice of the K matrix is performed.*<br><br>*If K is the matrix corresponding to the differential equation solution, the example so will be translated with:*<br><br>*1) A slicing with slicing triplet* <1,1,1> *for the last dimension in order to populate the* t *variable.*<br>*A slicing with slicing triplet* <2, 1, K.dimN>, *to populate the* y *matrix.*(2, 1, 2));
h=K(1)<br>disp(H)	h <- K[1]<br>print(h)	h = 5;<br>printf("%d\n", h);
error("abort")	Not implemented	printf("ERROR: abort, at line … of file …\n")
function Q=ICQ(x)<br>Q = exp(-0.5*x);	ICQ <- function(x){	static double Q;<br>double ICQ(int x){<br>double Q;

end	Q <- exp(-<br>0.5*x)<br>return(Q)<br>}	Q = exp(-0.5 * (double)x);<br>return Q;<br>}
isinf(x)	is.infinite(x)	isinf(x)
isnan(x)	is.nan(x)	isnan(x)
strcmp('a','b')	"a" == "b"	strcmp("a", "b")
zeros(5,1)	array(0, c(5,1))	int1dMatrix vec0;<br>static int vec0_vals[5] = {0, 0, 0, 0, 0};<br>vec0.matrix = vec0_vals;<br>vec0.dim1 = 5;<br><br>*As said above, the matrix can be statically allocated when the dimensions are known at compile-time, otherwise a calloc is performed*
ones(5,1)	array(1, c(5,1))	int1dMatrix vec0;<br>static int vec0_vals[5] = {1, 1, 1, 1, 1};<br>vec0.matrix = vec0_vals;<br>vec0.dim1 = 5;
NaN(5,1)	array(NaN, c(5,1))	int1dMatrix vec0;<br>static int vec0_vals[5] = {NAN, NAN, NAN, NAN, NAN};<br>vec0.matrix = vec0_vals;<br>vec0.dim1 = 5;
eye(2)	Not implemented	Int2dMatrix vec0;<br>static int vec0_vals[4] = {1, 0, 0, 1};<br>vec0.matrix = vec0_vals;<br>vec0.dim1 = 4;
diag	Not implemented	Algorithm written directly in place
unique([1,2])	unique(c(1,2))	v?Unique(…) an ad-hoc implementation of this MATLAB function in C shipped with the matrixLib library automatically included in translated programs.

A = size([2,5])	dim(c(2,5))	A.matrix[0] = vector1.dim1;<br>A.matrix[1] = vector1.dim2;<br>…
A =<br>length([1,2])	A <-<br>length(c(1,2))	A = max(vector1.dim1,<br>max(vector1.dim2, ….)…)
tic	tic <-<br>Sys.time()	tic = clock();<br><br>/*where tic is an environment<br>variable*/
E = toc	E<-toc<-<br>Sys.time()-tic<br><br>tic <-<br>Sys.time()	double e;<br>/*reset the tic variable*/<br>tic = clock();<br><br>e = ((double)clock() -<br>tic)/CLOCKS_PER_SEC;
interp1(x,v,xq,m<br>ethodName)	library(stats)<br>approx(x,v,xq,me<br>thod=methodName)<br>$y<br><br>*Only if the<br>MATLAB method is<br>“spline”:*<br><br>spline(x, v, xq)	dInterp(* xValues, int<br>xValuesLen, double* yValues,<br>int yValuesLen, double<br>xToBeInterpolated)<br><br>*where <type>Interp is a<br>matrixLib functions that,<br>iterating through yValues,<br>finds the nearest one. Thus is<br>always used the interpolation<br>mode “nearest”.*
subplot(3,2,1)<br>plot(x,y)<br>…<br>xlabel /<br>ylabel/legend/su<br>bfigure	par(mfrow=c(3,<br>2))<br>plot(x, y)<br><br>*Command title,<br>xlabel, ylabel,<br>legend,<br>subfigure are<br>ignored*	Warning, the plot commands in C<br>are not supported
data_array<br>=load(‘input.mat<br>’)	library(R.matlab<br>)<br>data_array <-<br>loadMat(“input.m<br>at”)<br><br><br>*Rlibrary<br>R.matlab stores*	CSV *data_array;<br>data_array=load(“input.csv”);<br><br><br>*Where load is an internal<br>function (library loadLib.c)<br>that loads the input file<br>(“input.csv”), where the csv<br>has been generated from the*

	data from .mat file in a list with attributes. Our function listToDataFrame converts the list to a data frame.	*mat-file with the script* mat2csv.m *, provided with the examples.*<br>*Csv values are statically stored to be accessible from every part of the program*
data_array =csvread('input.csv')	data_array <- loadCsv("input.csv")	CSV *data_array;<br>data_array=load("input.csv");<br><br>*Same behavior as for "load" function, but in MATLAB the csvread function doesn't expect any header.*
save(filename,variables)	*library(R.matlab)*<br>*writeMat(filename,variables)*	*Save functions in* saveLib.c *that allow to save several kinds of input in a file (arrays of both double and int, int and double and string variables). They are all based on* fprintf.
csvwrite(filename,K)	library(utils)<br>write.table(filename,K, sep=",", row.names=FALSE, col.names=FALSE)	*Save functions in* saveLib.c *that allow to save several kinds of input in a file (arrays of both double and int, int and double and string variables). They are all based on* fprintf.
exp(x)	exp(x)	exp(x)<br><br>*In the case of a matrix, the is performed cycling on very element with the functions* viExp *or* vdExp *in the library* MatrixLib.c
log(x)	log(x)	log(x)<br><br>*In the case of a matrix, the is performed cycling on very element with the functions* vLog *or* vdLog *in the library* MatrixLib.c
floor(a)	floor(a)	floor(a)

		In the case of a matrix, a cast to int is performed cycling on very element with the functions vdFloorO *in the library* MatrixLib.c
ceil(A)	ceiling(A)	ceil(x)<br><br>*In the case of a matrix, the ceil is performed cycling on very element with the function* vdCeilO *in the library* MatrixLib.c
mod (8,6)	8 %% 6	8 % 6
numel(K)	length(K)	K.dim1*K.dim2*…*K.dimN<br><br>*If K is a matrix, whereas if K is a sequence is computed as*<br><br>(K.end-K.start)/K.step
randn(5)	rnorm(5)	randn(0.0, 1.0, 1, 5)<br><br>*where randn is a function in the mkLib library that takes as input the mean and sigma of the normal distribution and then the matrix dimensions.*<br>*In order to generate the number, the mkl function* vdRngGaussian *is used*
rand	Not implemented	??randu
randi	Not implemented	??randi
randperm	Not implemented	Not implemented
A = [1 3]<br>sum(A)<br><br>A = [1 3 ; 4 2]<br>sum(A) *or* sum(A,1)<br><br>sum(A,2)	A=c(1,3)<br>sum(A)<br><br>A = rbind(c(1,2),c(3,4))<br>colSums(A)<br><br>rowSums(A)	??Sum(A.matrix, dim1, dim2, …)<br><br>*where sum is a function contained in the MatrixLib.c that iterates over the A.matrix elements and calculates their sum*
max(A)	max(A)	??Max(A) or ??Max(A)

		with vdMax *and* viMax *are two functions that iterate over all the A elements to find the maximum value*
min(A)	min(A)	??Min(A) or ??Min(A)<br><br>*with* vdMax *and* viMax *are two functions that iterate over all the A elements to find the maximum value*
pi	pi	*Replaced with its value (taken from the Math library in Java)*
lu(K)	library(Matrix) expand(lu(Matrix(K)))	Not implemented
schur(K)	library(Matrix) schur(Matrix(K))	Not implemented
expm(K)	library(Matrix) expm(Matrix(K))	Not implemented
clear	rm(list = ls())	Not implemented
hilb(4)	library(Matrix) Hilbert(4)	Not implemented
linspace(a,b,c)	seq(a,b,length=c)	dslice vector1 = {.start=a, .step=(b-a)/(c-1), .end=b, .linear=true};
sprintf(‘%0.6f\n’, x)	sprint(‘%0.6f\n’, x)	sprintf(‘%0.6f\n’, x)
log10(x)	log10(x)	log10(x)
strcat(str1,str2)	paste(str1,str2, sep=””)	Not implemented
fprintf(%4.2f meters or %8.3f, A1, A2)	fprintf(%4.2f meters or %8.3f, A1, A2)	Not implemented
pwd	getwd()	Not implemented

logspace(a,b,c)	library(pracma)<br>logspace(a,b,n=c)	dslice vector1 = {.start=a, .step=(b-a)/(c-1), .end=b, .linear=false};
find(X>2)	which(X>2)	Implemented by writing out directly the algorithm in place.
addpath('pathname')	*Additional files contained in the added paths are recognized and included directly by the front-end.*	*Additional files contained in the added paths are recognized and included directly by the front-end.*
setdiff	*Not implemented*	*??Setdiff*
union	*Not implemented*	*v?Union*
sort	*Not implemented*	*v?Sort*
isempty	*Not implemented*	*Through a set of dynamically generated *_IS_EMPTY macros*
round	*Not implemented*	*??round*
sin/cos/tan	*Not implemented*	??sin/??cos/??tan
sqrt	*Not implemented*	sqrt
power	*Not implemented*	power
kron	*Not implemented*	?KroneckerTensorProduct
abs	*Not implemented*	??abs
atan2	*Not implemented*	??atan2
sign	*Not implemented*	??sign
slice	*Not implemented*	?SparseSliceMatrix
norm	*Not implemented*	?MklMatrixNorm2
numel	*Not implemented*	v?SequenceDimension
sparse	*Not implemented*	?InitSparseScalarMatrix
triu	*Not implemented*	Algorithm translated in place
tril	*Not implemented*	Algorithm translated in place
repmat	*Not implemented*	?RepeatMatrix
eig	*Not implemented*	?MklEigenvalues
ismember	*Not implemented*	? ContainedInSlice/? ContainedInArray
reshape	*Not implemented*	v?Reshape

cumsum	*Not implemented*	v?CumSum
class	*Not implemented*	Nothing. It is a function that is used inside the translation process only to infer types.
break	*Not implemented*	break
continue	*Not implemented*	continue
nargin	*Not implemented*	Handled through an hidden variable for each function
TRUE	*Not implemented*	1
FALSE	*Not implemented*	0

*Supplementary Table 3 Mapping of MATLAB statements to target languages R and C. For matrix operation we use "double *" type in C target language, as it can be safely translated to Sundials NVector through "N_Vmake_Serial" Sundials function.*

6.1 NUMERICAL ODE SOLVER METHODS

In Supplementary Table 4 and Supplementary Table 5, we report the mapping of MATLAB ODE solver methods to corresponding methods in R deSolve and C Sundials, respectively. Detailed information about the syntax of these methods is also reported, as derived from the corresponding documentation. Where possible, perfect correspondence has been kept, as for ode23 and ode45 from MATLAB to R. In other cases, methods are chosen in order to have at least a partial correspondence regarding the type of algorithm, while at the same time giving satisfying results in terms of performance and accuracy.

In the following CV_ADAMS stands for the Adams-Moulton linear multistep method, whereas CD_BDF stands for BDF linear multistep method. For what concerns the non-linear system solution, CV_FUNCTIONAL stands for nonlinear system solution through functional iterations and CV_NEWTON for nonlinear system solution through Newton iteration.

MATLAB	Sundials (C)
ode45	%Initialization code%<br>Void * cvodemem = CVodeCreate(CV_BDF, CV_NEWTON)[1]
ode45(odefun,tspan,y0,options)	%Execution Code%<br><br>N_Vector y = N_VNew_Serial(y_len);<br>for (k = ZERO; k < y_len; k++){<br>Ith(s->y, k) = y[k];<br>}<br>CVodeInit(cvode_mem, f, T0, y);<br>CVodeSStolerances(cvode_mem, RTOL, ATOL);<br>CVDense(cvode_mem, y_len);<br>CVodeSetMaxStep(cvode_mem, MAX_STEP);<br>CVodeSetUserData(cvode_mem, USERDATA);<br>CVodeSetInitStep(cvode_mem, INITIAL_STEP);<br>CVodeSetMaxOrder(cvode_mem, MAX_ORDER)<br><br>CVode(cvode_mem,T1, y, &t, CV_NORMAL);
ode23	%Initialization code%<br>CVodeCreate(CV_BDF, CV_NEWTON)
ode23(odefun,tspan,y0,options)	Execution as above
ode113	%Initialization code%<br>CVodeCreate(CV_BDF, CV_NEWTON)
ode113(odefun,tspan,y0,options)	Execution as above
ode15s	%Initialization code%<br>CVodeCreate(CV_BDF, CV_NEWTON)
ode15s(odefun,tspan,y0,options)	Execution as above
ode23s	%Initialization code%<br>CVodeCreate(CV_BDF, CV_NEWTON)
ode23s(odefun,tspan,y0,options)	Execution as above

Supplementary Table 4 MATLAB ODE-solver method and their translation to C Sundials

[1] As a first attempt, we tried to translate the non-stiff methods (as ode45, ode23 and ode113) with the non-stiff sundials method built with the command CVodeCreate(CV_ADAMS, CV_FUNCTIONAL), but this method caused integration times much longer than the ones we got with the CV_BDF, CV_NEWTON method.

MATLAB	deSolve (R)
ode45	ode45
ode45(odefun,tspan,y0,options)	ode(y, times, func, parms, rtol = 1e-6, atol = 1e-6, verbose = FALSE, tcrit = NULL, hmin = 0, hmax = NULL, hini = hmax, ynames = TRUE, method = “ode45”, maxsteps = 5000, dllname = NULL, initfunc = dllname, initpar = parms, rpar = NULL, ipar = NULL, nout = 0, outnames = NULL, forcings = NULL, initforc = NULL, fcontrol = NULL, events = NULL, ...)
ode23	ode23
ode23(odefun,tspan,y0,options)	ode(y, times, func, parms, rtol = 1e-6, atol = 1e-6, verbose = FALSE, tcrit = NULL, hmin = 0, hmax = NULL, hini = hmax, ynames = TRUE, method = “ode23”, maxsteps = 5000, dllname = NULL, initfunc = dllname, initpar = parms, rpar = NULL, ipar = NULL, nout = 0, outnames = NULL, forcings = NULL, initforc = NULL, fcontrol = NULL, events = NULL, ...)
ode113	lsoda
ode113(odefun,tspan,y0,options)	lsoda(y, times, func, parms, rtol = 1e-6, atol = 1e-6, jacfunc = NULL, jactype = "fullint", rootfunc = NULL, verbose = FALSE, nroot = 0, tcrit = NULL, hmin = 0, hmax = NULL, hini = 0, ynames = TRUE, maxordn = 12, maxords = 5, bandup = NULL, banddown = NULL, maxsteps = 5000, dllname = NULL, initfunc = dllname, initpar = parms, rpar = NULL, ipar = NULL, nout = 0, outnames = NULL, forcings = NULL, initforc = NULL, fcontrol = NULL, events = NULL, lags = NULL,...)
ode15s	lsodes
ode15s(odefun,tspan,y0,options)	lsodes(y, times, func, parms, rtol = 1e-6, atol = 1e-6, jacvec = NULL, sparsetype = "sparseint", nnz = NULL, inz = NULL, rootfunc = NULL, verbose = FALSE, nroot = 0, tcrit = NULL, hmin = 0, hmax = NULL, hini = 0, ynames = TRUE, maxord = NULL, maxsteps = 5000, lrw = NULL, liw = NULL, dllname = NULL, initfunc = dllname, initpar = parms, rpar = NULL, ipar = NULL, nout = 0, outnames = NULL, forcings=NULL, initforc = NULL, fcontrol=NULL, events=NULL, lags = NULL, ...)

ode23s	lsodes
ode23s(odefun,tspan,y0,options)	lsodes(y, times, func, parms, rtol = 1e-6, atol = 1e-6, jacvec = NULL, sparsetype = "sparseint", nnz = NULL, inz = NULL, rootfunc = NULL, verbose = FALSE, nroot = 0, tcrit = NULL, hmin = 0, hmax = NULL, hini = 0, ynames = TRUE, maxord = NULL, maxsteps = 5000, lrw = NULL, liw = NULL, dllname = NULL, initfunc = dllname, initpar = parms, rpar = NULL, ipar = NULL, nout = 0, outnames = NULL, forcings=NULL, initforc = NULL, fcontrol=NULL, events=NULL, lags = NULL, ...)

Supplementary Table 5 MATLAB ODE-solver method and their translation to R deSolve.

6.2 ODE NUMERICAL SOLVER PARAMETERS

In Supplementary Table 6, we set side by side the ODE solver parameters for MATLAB, C library *Sundials* and R library *deSolve*. When parameters correspond, they are reported in the same row. Lack of a correspondent parameter is marked as NA. Default values are reported. In the case of *deSolve*, the list of integration methods accepting that parameter is also reported.

	MATLAB	Sundials (C)	deSolve (R)	deSolve (R) methods accepting the parameter
**Relative Tolerance.** Relative error tolerance. This tolerance measures the error relative to the magnitude of each solution component.	RelTol	CVodeSStolerances(cvode mem, reltol, abstol);	rtol	lsoda<br>lsodes<br>ode45<br>ode23
	1e-3	1e-6	1e-6	
**Absolute Tolerance.** Absolute error tolerance.	AbsTol	CVodeSStolerances(cvode mem, reltol, abstol);	atol	lsoda<br>lsodes<br>ode45<br>ode23
	1e-6	1e-8	1e-6	
**Non Negative.** The scalar or vector selects which solution components must be nonnegative.	NonNegative	NA	NA	
	None	-	-	
**Output Function.** Output function, the ODE solver calls the output function after each successful time step.	OutputFcn	NA	NA	
	None	-	-	
**Output Selection.** Component selection for output function.	OutputSel	NA	NA	
	None	-	-	

**Refine.** Solution refinement factor, a scalar that specifies a factor by which the number of output points should increase in each step.	Refine	NA	NA	
	1	-	-	
**Solver statistics.** When activated, the solver displays information after completing the solution.	Stats	NA*	verbose	lsoda lsodes ode45 ode23
	off	off	FALSE	
**Initial Step.** Suggested initial step size.	InitialStep	CVodeSetInitStep	hini	lsoda lsodes ode45 ode23
	If you do not specify an initial step size, then the solver bases the initial step size on the slope of the solution at the initial time point, tspan(1).	Estimated by solver. See Sundials doc for the algorithm used.	Estimated by solver	
**Events.** Events to be taken into account during the IVP solving.	Events	NA	NA	
	None	-	-	

**Jacobian.** Specifies the Jacobian matrix, or the function used to compute it.	Jacobian	CVDVIsSetJacFn[2]	jacfunc (function returning Jacobian matrix is needed)	lsoda
		By default, cvdls uses an internal	NULL	

[2] This functionality has not been implemented in our translation yet.

		difference quotient function for dense and band matrices.		
**Jacobian Pattern.** Jacobian sparsity pattern.	JPattern	NA	inz	lsoda<br>lsodes
	Non Negative	-	NULL	
**Jacobian vector** Function that computes a column of the Jacobian	NA	NA	jacvect	lsodes
	-	-	NULL	
**Sparsity** Sparsity structure of the Jacobian	NA	NA	sparsetype =	lsodes
	-	-	“sparseint”	
**Nonzero elements** Number of nonzero elements in the Jacobian	NA	NA	nnz	lsodes
	-	-	NULL	
**Vectorized.** Toggle to say whether the function accepts and returns vectors for the second argument.	Vectorized	NA	NA	
	Off	-	-	
**Mass Matrix**. The ODE solvers can solve problems containing a mass matrix.	Mass	NA	NA	
	None	-	-	
**Max step size.** Sets an upper bound on the size of any step taken by the solver.	MaxStep	CVodeSetMaxStep	hmax	lsoda<br>lsodes<br>ode45<br>ode23
	0.1*Abs(t0-tf)	None	Largest difference in times array.	
**MvPattern**. State dependence of mass matrix.	MvPattern	NA	NA	
	None	-	-	
**MassSingular.**	MassSingular	NA	NA	

Singular mass matrix toggle.	maybe	-	-	
**Initial Slope.** Consistent initial slope.	InitialSlope	NA	NA	
	Vector of zeros	-	-	
**Max Order.** Use this option to specify the maximum order used in the numerical differentiation formulas (NDFs) or backward differentiation formulas (BDFs).	MaxOrder	CVodeSetMaxOrd	maxord/ maxordern / maxorders	lsoda(maxordern/ maxorders) lsodes(maxord)
	5	12 for Adams, 5 for BDF.	12 for non-stiff, 5 for stiff methods	
**BDF.** Toggle that specifies whether using the default numerical differentiation formulas (NDFs) or BDFs.	BDF	NA	NA	
	off	True	True	
**Jacobian structure** The Structure of the Jacobian	NA	NA	jactype	lsoda
			"fullint"	
**Root of function** An R function that computes the function whose root has to be estimated	NA	CVodeRootInit(cvode_mem, n_root, g)	rootfunc	lsoda lsodes ode45 ode23
		NA	NULL	
**Number of roots** The number of constraints function whose roots are desired during the integration	NA	Specified in the last parameter of the function CVodeRootInit	nroot	lsoda lsodes ode45 ode23
		NA	0	
**Limit time** Time limit for integration	NA	CVodeSetStopTime	tcrit	lsoda lsodes ode45 ode23
		NA	NULL	

**Minimum step** Minimum value of the integration stepsize	NA	CVodeSetMinStep	hmin	lsoda lsodes ode45 ode23
		0	0	
**State variable names** Logical, if names of state variables are not passed to the function	NA	NA	ynames	lsoda lsodes ode45 ode23
			TRUE	
**Jacobian bands above diagonal** Number of non-zero bands above diagonal	NA	NA	bandup	lsoda
			NULL	
**Jacobian bands below diagonal** Number of non-zero bands below diagonal	NA	NA	banddown	lsoda
			NULL	
**Maximum steps** Maximum number of step per output interval	NA	CVodeSetMaxNumSteps	maxsteps	lsoda lsodes ode45 ode23
		500	5000	
**Shared library** String giving the name of the shared library with compiled functions	NA	NA	dllname	lsoda lsodes ode45 ode23
			NULL	
**Function name in shared library** Name of the initialization function as provided in ddlname	NA	NA	initfunc	lsoda lsodes ode45 ode23
			dllname	
**Vector of double values for shared library** Vector with double precision values passed to dllname	NA	NA	rpar	lsoda lsodes ode45 ode23
			NULL	
**Vector of integer values**	NA	NA	ipar	lsoda lsodes ode45

**for shared library** Vector with integer precision values passed to dllname				ode23
			NULL	
**Output variables in shared library** number of output variables calculated in the compiled function in the shared library	NA	NA	nout	lsoda lsodes ode45 ode23
			0	
**Names of output variables in shared library** Names of output variables calculated in the compiled function	NA	NA	outnames	lsoda lsodes ode45 ode23
			NULL	
**Forcing function** List with the forcing function data sets	NA	NA	forcings	lsoda lsodes ode45 ode23
			NULL	
**Name of forcing function** Name of the forcing initialization function	NA	NA	initforc	lsoda lsodes ode45 ode23
			NULL	
**Parameters for forcing function** List of control parameter for forcing function	NA	NA	fcontrol	lsoda lsodes ode45 ode23
			NULL	
**Events** Matrix or data frame that specifies events	NA	NA	events	lsoda lsodes ode45 ode23
			NULL	
**Time lags** List that specifies time lags	NA	NA	lags	lsoda lsodes
	NA		NULL	
**Error File Specifier** Pointer to an error file	NA	CVodeSetErrFile	NA	
		stderr		
**Error Handler Function**	NA	CVodeSetErrHandlerFn	NA	

Specifies the function to be used in handling error messages		Internal function	
**User Data** Specifies the user data block to pass external/environment variables to the function	NA	CVodeSetUserData<br>NA	NA
**Maximum warning number** Specifies the maximum warning number to be issued by the solver in one step	NA	CVodeSetMaxHnilWarns<br>10	NA
**Maximum number of error test failures** Specifies the maximum error test failures permitted in attempting one step	NA	CVodeSetMaxErrTestFails<br>7	NA
**Nonlinear solver iteration maximum number** Specifies the maximum numbers of nonlinear solver iterations permitted per step	NA	CVodeSetMaxNonlinIters<br>3	NA
**Convergence failure maximum number** Specifies the maximum number of nonlinear convergence	NA	CVodeSetMaxConvFails<br>10	NA

failures permitted during one step				
**Coefficient in the nonlinear convergence test**	NA	CVodeSetNonlinConvCoef	NA	
		0.1		
**Nonlinear iteration type** Resets the nonlinear solver iteration type to the specified one (CV_NEWTON or CV_FUNCTIONAL)	NA	CVodeSetIterType	NA	
		The iteration type previously specified in CVodeCreate		
**Root directions** Specifies the direction of zero-crossing to be found and return (for rootfinding)	NA	CVodeSetRootDirection	NA	
		both		
**Root finding warnings enabled** Disables issuing warnings if some root function appears to be 0 at the beginning of the integration	NA	CVodeSetNoInactiveRootWarn	NA	
		NA		

Supplementary Table 6 ODE solver parameters of MATLAB, Sundials and deSolve describing correspondences between different languages and default values when available
