# Supplementary material for: QSPcc reduces bottlenecks in computational model simulations
Source: Commun Biol. 2021 Sep 1;4:1022. doi: 10.1038/s42003-021-02553-9 (PMC8410852; doi:10.1038/s42003-021-02553-9)
Supplement: Supplementary file 2 — Reporting Summary [file 42003_2021_2553_MOESM2_ESM.pdf]

nature research

Corresponding author(s): Rosario Lombardo

Last updated by author(s): Jun 15, 2021

Reporting Summary

Nature Research wishes to improve the reproducibility of the work that we publish. This form provides structure for consistency and transparency in reporting. For further information on Nature Research policies, see our Editorial Policies and the Editorial Policy Checklist.

Statistics

For all statistical analyses, confirm that the following items are present in the figure legend, table legend, main text, or Methods section.

n/a	Confirmed	
☐	☒	The exact sample size (n) for each experimental group/condition, given as a discrete number and unit of measurement
☒	☐	A statement on whether measurements were taken from distinct samples or whether the same sample was measured repeatedly
☐	☒	The statistical test(s) used AND whether they are one- or two-sided<br>*Only common tests should be described solely by name; describe more complex techniques in the Methods section.*
☒	☐	A description of all covariates tested
☒	☐	A description of any assumptions or corrections, such as tests of normality and adjustment for multiple comparisons
☐	☒	A full description of the statistical parameters including central tendency (e.g. means) or other basic estimates (e.g. regression coefficient) AND variation (e.g. standard deviation) or associated estimates of uncertainty (e.g. confidence intervals)
☒	☐	For null hypothesis testing, the test statistic (e.g. F, t, r) with confidence intervals, effect sizes, degrees of freedom and P value noted<br>*Give P values as exact values whenever suitable.*
☒	☐	For Bayesian analysis, information on the choice of priors and Markov chain Monte Carlo settings
☒	☐	For hierarchical and complex designs, identification of the appropriate level for tests and full reporting of outcomes
☒	☐	Estimates of effect sizes (e.g. Cohen's d, Pearson's r), indicating how they were calculated

Our web collection on statistics for biologists contains articles on many of the points above.

Software and code

Policy information about availability of computer code

Data collection	The data in this work can be considered as the list of mathematical models of biological processes published in other peer-reviewed articles. It is accurate to say we used PubMed to retrieve such works.
Data analysis	The software we used to create QSPcc include: ANTLR version 3 and 4 to understand Matlab and R languages, openMPI for parallel computation optimization, Sundials from 2.7 and 3.0 up to 5.7.0 for integration algorithms plus a number of other miscellanea packages such as TCMALLOC and MATLAB libraries for MEX integration and several private integrations with Intel Math Kernel Libraries for hardware optimization. gcc version 5.4.0 20160609 was used to create efficient binary code from auto-generated C source. We also used MATLAB R2021a 64bit and R 3.4.3 linux to run the mathematical models of biological processes described in the text.

For manuscripts utilizing custom algorithms or software that are central to the research but not yet described in published literature, software must be made available to editors and reviewers. We strongly encourage code deposition in a community repository (e.g. GitHub). See the Nature Research guidelines for submitting code & software for further information.

Data

Policy information about availability of data

All manuscripts must include a data availability statement. This statement should provide the following information, where applicable:

- Accession codes, unique identifiers, or web links for publicly available datasets
- A list of figures that have associated raw data
- A description of any restrictions on data availability

QSPcc is released under the BSD 3-clause license, publicly available from the GitHub repository https://github.com/cosbi-research/QSPcc. At the same location are available all information to access the tests and docker files

Field-specific reporting

Please select the one below that is the best fit for your research. If you are not sure, read the appropriate sections before making your selection.

☒ Life sciences ☐ Behavioural & social sciences ☐ Ecological, evolutionary & environmental sciences

For a reference copy of the document with all sections, see nature.com/documents/nr-reporting-summary-flat.pdf

Life sciences study design

All studies must disclose on these points even when the disclosure is negative.

Sample size	No biological samples were used. The sample may be considered the mathematical models of the biological processes whose executions were replicated 5 times to asses the average value of the effective result.
Data exclusions	We extensively searched the literature using databases such as PubMed and Google Scholar for modeling papers describing a MATLAB implementation. From the results we identified, we considered only those satisfying these criteria: 1) the source was available as a set of MATLAB scripts, 2) it was of a significant dimension, 3) the code ran as-is on MATLAB. The same approach was used to identify other tools compared in the study for which we simply required to be available and working. When those 3rd party tools were not able to run on 3rd party models we declared so.
Replication	Reproducibility is guaranteed by automatic testing of all computational models. Multiple executions, considered as replicates, were performed to assess the trend.
Randomization	For ODE models, randomization was not an issue due to the deterministic nature of the application. Five replicate runs were considered to take into account any machine-specific change like temporary slow-downs. For stochastic models the replication approach showed the dynamic biological tendency confirming the agreement among the model replicate runs.
Blinding	No blinding is applicable in this study.

Reporting for specific materials, systems and methods

We require information from authors about some types of materials, experimental systems and methods used in many studies. Here, indicate whether each material, system or method listed is relevant to your study. If you are not sure if a list item applies to your research, read the appropriate section before selecting a response.

Materials & experimental systems

n/a	Involved in the study	
☒	☐	Antibodies
☒	☐	Eukaryotic cell lines
☒	☐	Palaeontology and archaeology
☒	☐	Animals and other organisms
☒	☐	Human research participants
☒	☐	Clinical data
☒	☐	Dual use research of concern

Methods

n/a	Involved in the study	
☒	☐	ChIP-seq
☒	☐	Flow cytometry
☒	☐	MRI-based neuroimaging
